# Study on the Low Plastic Behavior of Expansion Deformation of Austenitic Stainless Steel

**DOI:** 10.3390/ma17091955

**Published:** 2024-04-23

**Authors:** Chenglei Wan, Wei Wu, Xuedi Zhang, Lulu Zhang, Kaihong Song

**Affiliations:** School of Materials Science and Engineering, Hebei University of Technology, Tianjin 300401, China; 13833844540@163.com (C.W.); 13821914840@163.com (W.W.); 17860391815@163.com (X.Z.); 15231730346@163.com (L.Z.)

**Keywords:** TWIP steel, expansion, twins, low plasticity

## Abstract

The plastic deformation of TWIP steel is greatly inhibited during the expansion process. The stress–strain curves obtained through expansion experiments and observations of fracture morphology confirmed the low plastic behavior of TWIP steel during expansion deformation. Through an analysis of the mechanical expansion model, it was found that the expansion process has a lower stress coefficient and a faster strain rate than stretching, which inhibits the plasticity of TWIP steel during expansion deformation. Using metallographic microscopy, transmission electron microscopy, and EBSD to observe the twin morphology during expansion deformation and tensile deformation, it was found that expansion deformation has a higher twin density, which is manifested in a denser twin arrangement and a large number of twin deliveries in the microscopic morphology. During the expansion deformation process, dislocation slips are hindered by twins, the free path of the slips is reduced, and dislocations accumulate significantly. The accumulation area is the initial point of crack expansion. The results show that the significant dislocation accumulation caused by the delivery of a large number of twins under expansion deformation is the main reason for the decrease in the plasticity of TWIP steel.

## 1. Introduction

Expansion tubes are an important emerging technology in the drilling field. This technology can be used to repair production casing damage caused by sucker rod damage or downhole corrosion, as well as to repair old well production casing [1,2,3]. In order to adapt to developments in the oil and gas industry, especially regarding underground temperatures, it is of great significance to develop high-strength, high-plasticity, and corrosion-resistant materials for expandable steel pipes [4,5,6,7]. At present, 316L stainless steel is mostly used as the expanding pipe material, as it meets the technical requirements for shallow oil wells. However, for deep oil and gas wells (2000–4000 m) and ultra-deep wells (4000–10,000 m), there are potential safety risks [8]. The performance of expandable tube materials limits the development of expandable tube technology [9]. Exploring high-quality steel as an expanding material is of great significance. Twinning-induced plasticity steel, usually called TWIP steel, has a stable austenitic structure at room temperature; high elasticity, strength, and elongation; and good low-temperature properties and strain hardening properties. These properties have attracted both domestic and widespread attention in global high-tech fields such as automobiles and petroleum [10,11,12,13].

The deformation mechanism of TWIP steel is linked to its low stacking fault energy, which causes dislocation to be hindered by the deformation twin boundaries, subsequently causing the dislocations to wind and accumulate around the twin boundaries [14,15]. Twin boundaries play a role similar to grain boundaries, so the formation of twin boundaries during deformation refines the grains; this is the main reason for the high strength and high plasticity of TWIP steel and is called the dynamic Hall–Page effect [16,17,18]. At the same time, the formation of deformation twins leads to the accumulation of a certain amount of pre-strain, which improves the plasticity of the material [19]. Bruno C. De Cooman et al. [20] summarized the development of TWIP steel history, successfully summarized the deformation mechanism of TWIP steel, and further proposed the main problems faced by TWIP steel today: (1) Uncertainty in the assessment of the contribution of various mechanisms to strain strengthening. (2) It is impossible to directly and accurately observe twins through experimental evolution. (3) The role of carbon in TWIP steel is unclear. CD Hondt et al. [21] conducted on-site uniaxial tension and tension compression tests on Fe 22Mn-0.6C TWIP steel under SEM and AFM and found that under a fixed plastic strain range within a range, during the cyclic hardening process, the number of twinned grains and the average number of active twins per grain both increase. The number of twins in the largest grains in the plastic strain range that can be accommodated becomes smaller and smaller.

Using TWIP steel to prepare expandable pipes not only improves their performance but also improves work safety and expands the scope of their applications. Additionally, it provides competitive advantages and cost savings for expandable pipe manufacturers. Therefore, TWIP steel has good economic and social benefits and broad development prospects [22,23,24].

However, the reason for the significant difference in the expansion and tensile deformation plasticity of TWIP steel remains unclear. This study aimed to clarify the distinction between the expansion and tensile processes of TWIP steel, which is the primary cause of its low expansion plasticity. These findings shall provide further details on the mechanism of the plastic deformation of TWIP steel and provide a theoretical basis for the selection and application of expandable tube materials.

## 2. Experimental Materials and Methods

### 2.1. Mechanical Property Test

**Figure 1:**
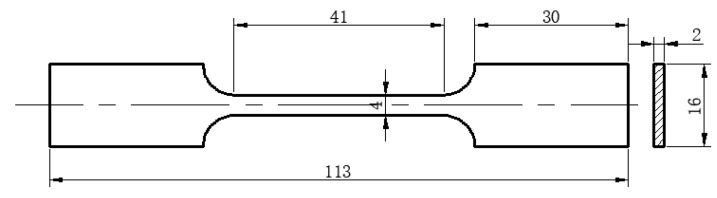
Tensile specimen.

The material for this test was Fe-Mn-Si-Al series rolled TWIP steel with an austenite structure at room temperature. The material composition is shown in Table 1. In order to remove the rolled structure, the pipe was placed in a high-temperature furnace and subjected to solution treatment of 1050 °C for 30 min and then cooled with water. The TWIP steel pipe used in the test had an inner diameter of 50 mm and a wall thickness of 3 mm. In order to obtain a specimen for the tensile test, a 20 mm strip was cut along the axial direction of the pipe. After simple flattening, the tensile specimen was cut along the axial direction of the pipe, as shown in Figure 1, according to GB/T228.1-2021 [25]. The tensile rate used in the tensile test was 3 mm/min. The long pipe was cut into 100 mm short pipes for expansion tests.

**Figure 2:**
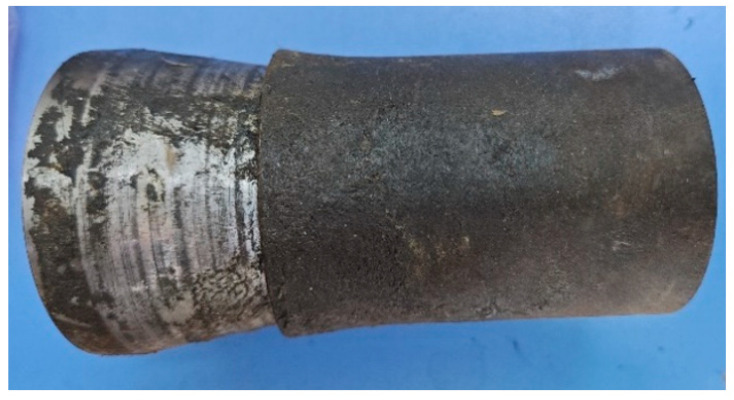
Expansion cone and pipe.

In the expansion test, an expansion cone was compressed into the pipe using the testing machine to initiate the expansion process. This test used a single expansion. The expansion cone material was 42CrMo and the expansion cone angle was 15°. By using expansion cones of different sizes, the pipes are subjected to different expansion rates. The moving speed of the expansion cone was 3 mm/min. The pipe and expansion cone used in the expansion test are shown in Figure 2. Before the test, molybdenum disulfide grease was evenly applied on the surface of the expansion cone and the inner wall of the pipe to reduce the friction coefficient.

The expansion and uniaxial tensile tests were both conducted using a WDW-200Y universal testing machine. After the test, force and displacement data were recorded.

In the expansion test, the expansion cone was compressed using the testing machine and was placed in the pipe to initiate expansion. This test involved a single expansion with an expansion cone angle of 15°. The expansion cone movement rate and stretching rate were both 3 mm/min.

### 2.2. Microstructure Observation

After completing the expansion and tensile tests, samples with expansion rates of 6% and 10% were selected and 5 × 5 mm block metallographic samples were taken from the deformation zone. Fracture samples were taken from expansion specimens with an expansion rate of 15% and tensile specimens with a tensile rate of 50%. The sample block was ground and polished, and then the polished surface was chemically etched in a 4% nitric acid alcohol solution for 10 to 15 s. An optical microscope (OM) was used to observe the structure, and SEM was used to observe the surfaces of the tensile and expansion fractures. TEM experiments were conducted on expanded specimens and tensile specimens at different stages. Additionally, electron backscattered diffraction (EBSD) was used to compare the differences between expanded and stretched structures, as well as the changes in the tissue during the expansion process.

## 3. Experimental Results and Discussion

### 3.1. Mechanical Performance Analysis

After the uniaxial tensile test, force and displacement data were recorded. First, the engineering stress and strain were calculated through Equations (1) and (2), and then the true stress and strain were obtained through Equations (3) and (4).

(1) $$\sigma =\frac{F}{A}$$

(2) $$\varepsilon =\frac{X}{L}$$

(3) S=σ(1+ε)

(4) e=Ln(1+ε)

In these formulae, *σ* is the engineering stress; *F* is the force obtained by the test (N); *A* is the cross-sectional area at the gauge length (mm^2^); *ε* is the engineering strain; *S* is the true stress (MPa); and *e* is the true strain.

It should be noted that the cross-sectional area of the pipe subjected to force deformation during the expansion process is uncertain, and the topic discussed in this article is the low plastic behavior of TWIP steel during the expansion process, so the crack width is used as the deformation in the stress calculation process and the cross-sectional area, which does not affect the calculation of strains. After the 15% expansion test, a 2 mm-long crack was found on the sample. Therefore, a width of 2 mm and a cross-sectional area of 6 mm^2^ were used in the calculation. Since the test determines the downward movement of the expansion cone, not the circumferential deformation of the pipe, the calculation cannot be performed directly. First, the experimental result must be calculated and converted into the circumferential deformation of the pipe through Equation (5). This calculation can be performed by replacing X in Equation (2) with Δ*C* and *L* with the pipe circumference *C*.

(5) ∆C=2πXtan15°

**Figure 3:**
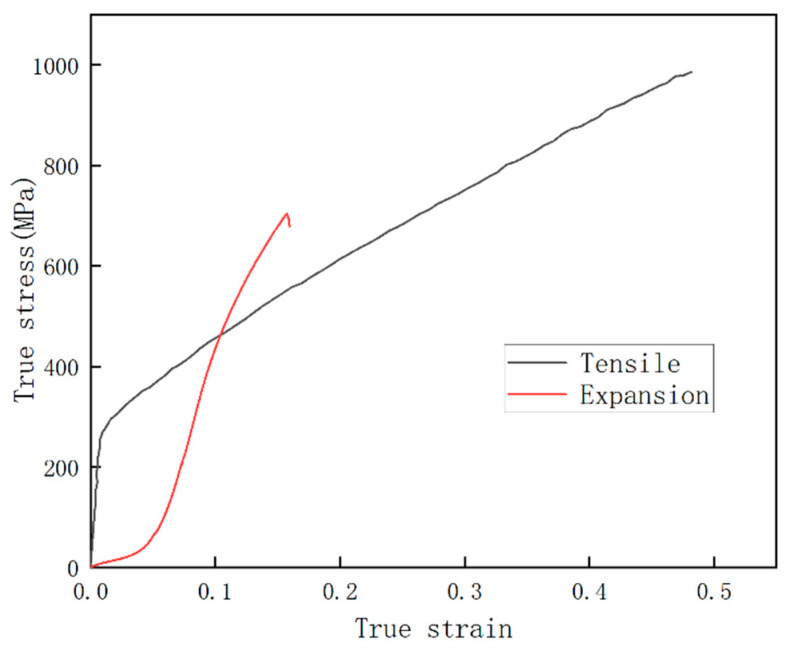
Tensile and expansion true stress–strain curves of TWIP steel.

Figure 3 shows the calculated tensile and expansion true stress–strain curves. It can be observed that there are obvious differences between the expansion and the tensile deformation curves. The expansion curve of TWIP steel does not exhibit an obvious elastic deformation stage, indicating that the steel undergoes plastic deformation from the beginning of the expansion process and does not undergo obvious elastic deformation. The TWIP steel pipe broke when the expansion strain was 15%, while in the tensile test, the strain did not cause fracture until it reached 50%. After comparing the expansion and tensile curves, it was found that expansion deformation greatly inhibits the plastic deformation ability of TWIP steel.

### 3.2. Fracture Morphology

**Figure 4:**
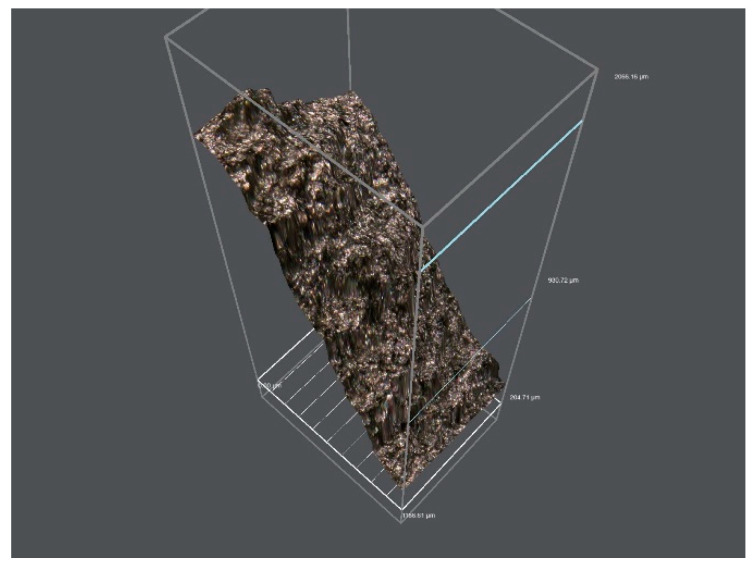
3D view of pipe expansion fracture crack surface.

Figure 4 is a 3D view of the crack surface of the expansion fracture of the pipe. It can be seen from Figure 3 that the crack surface is undulating and rough. The crack surface shows a slope shape as a whole, and some tiny areas inside show a waterfall shape with a steeper slope.

**Figure 5:**
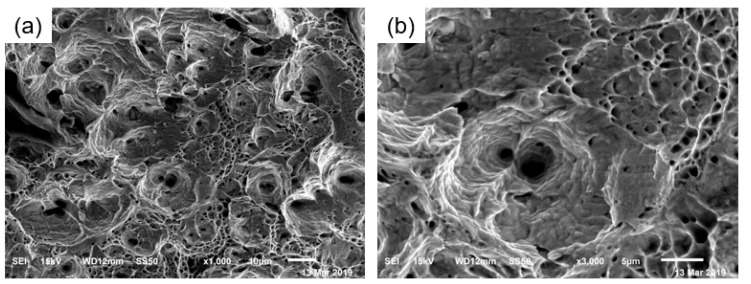
Tensile fracture morphology of TWIP steel: (**a**) ×1000; (**b**) ×3000.

**Figure 6:**
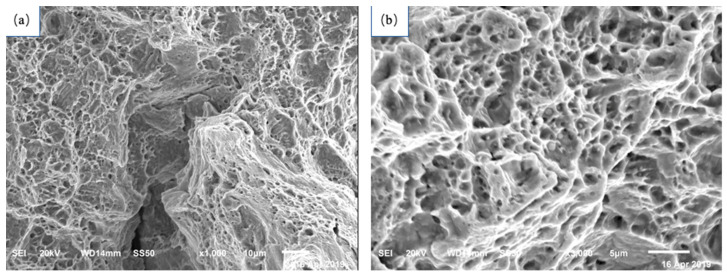
TWIP steel expansion fracture morphology (**a**) ×1000; (**b**) ×3000.

Figure 5 shows the uniaxial tensile fracture morphology of TWIP steel. The steel exhibits ductile fracture with a large number of unevenly sized dimples. Figure 6 is a scan of the crack at the fracture surface of the TWIP steel pipe. It can be observed from Figure 4 that the fracture surface of the steel pipe has an equiaxial dimpled structure. The size of the dimples is relatively uniform, tapered along the depth direction, and there are many secondary dimples and micron-scale dimples within the larger dimples. During the deformation process, a large number of twins are formed in TWIP steel. Twin boundaries can hinder the movement of dislocations to a certain extent, and micropores are nucleated at the twin boundaries under the interaction of deformation twins and dislocations. As deformation progresses, the resulting stress concentration causes the micropores to continue to grow and connect with other micropores, resulting in the occurrence of ductile fractures until the material breaks.

In the left area of Figure 6a, large-area platforms are visible, which have flat mirror-like surfaces. Consequently, the fracture form in this area is cleavage fracture. Most of these can be found in Figure 6b. There are second-phase particles in the small dimples. The expansion fracture of the TWIP steel pipe is a mixed fracture mode, consisting of dimple-like fractures and a very small amount of cleavage fractures, which behave overall as a rapid ductile fracture.

### 3.3. Deformation Twin Configuration

**Figure 7:**
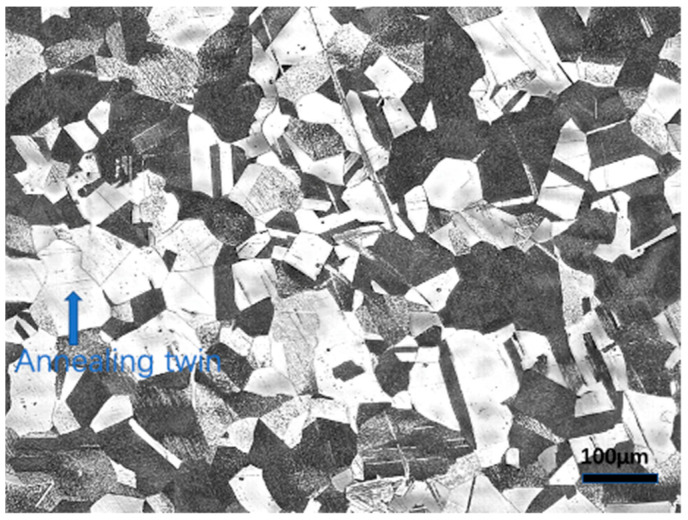
Metallographic structure of TWIP steel after solid solution treatment.

As shown in Figure 7, after heat treatment, the distribution of grains in TWIP steel is uniform; the structure has been eliminated after rolling and annealing twins are generated within the grains. The metallographic photographs show the different levels of brightness and darkness caused by variations in the grain orientation and shape.

**Figure 8:**
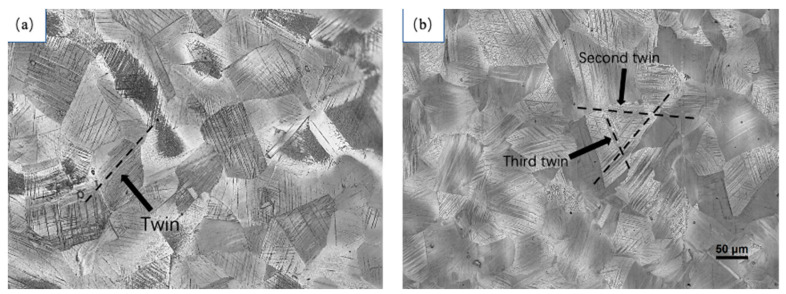
Tensile and expansion fracture metallographic structure: (**a**) tensile; (**b**) expansion.

Figure 8 shows the metallographic structure after fracture in two deformation modes: tensile and expansion. Figure 8a shows the tensile fracture structure, while Figure 8b shows the expansion fracture structure. Both deformation methods lead to the generation of a large number of twins within the austenite grains. These twins often run through the entire grains and are hindered by the boundaries between grains. The distribution directions of twin planes between different grains are different.

**Figure 9:**
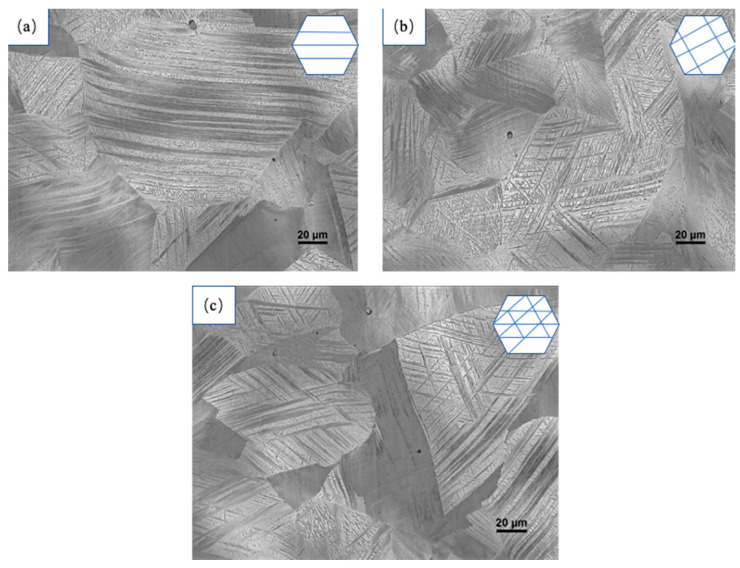
TWIP steel metallographic structure observed at different expansion rates: (**a**) 6%; (**b**) 10%; (**c**) 15%.

Upon comparing the twin configurations after tensile fracture and expansion fracture, it was observed that the twins after tensile fracture consist mainly of a large number of primary twins and a small amount of secondary twins. After expansion fracture, the twins are arranged very densely, and the delivery between twins is more serious. These deliveries are caused by primary twins, secondary twins, and tertiary twins. These tertiary twins are hindered by the twin boundaries of primary twins and secondary twins forming between the two twin interfaces. Figure 9 shows the metallographic structure observed in samples with expansion rates of 6%, 10%, and 15%, respectively. As the expansion stage progresses, the number of twins increases, and at the same time, the configuration of the twins changes significantly. When the expansion rate is 6%, there are a large number of primary twin planes distributed in parallel. As the expansion stage proceeds, secondary twin delivery begins to form until tertiary twin delivery occurs when fracture occurs.

### 3.4. Mechanical Analysis of Expansion Deformation of TWIP Steel

**Figure 10:**
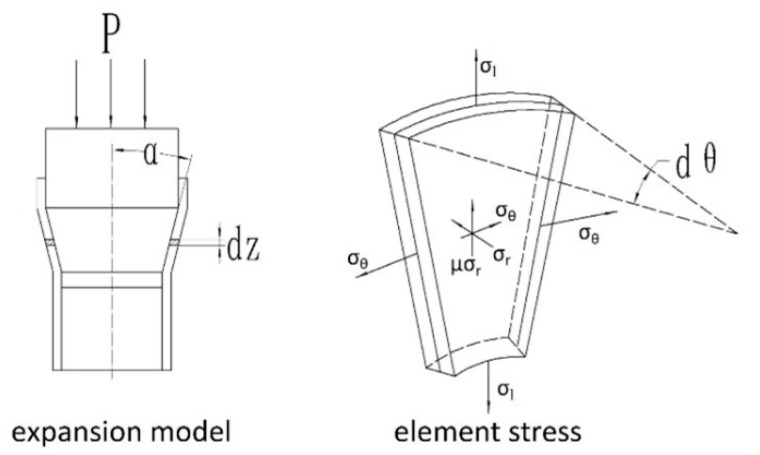
Mechanical model of expansion and deformation.

The experimental results in Figure 3 show the low plastic behavior of TWIP steel during the expansion process. The micromorphology of the fracture surface also confirms this low plastic behavior of TWIP steel. The mechanical behavior of materials is not only related to the performance attributes of the material itself, but also to different stress and deformation modes, which are also the main factors affecting material deformation. Through the analysis of mechanical behavior, it was found that the complex stress behavior of the expansion process is the main reason for the low plastic behavior of TWIP steel. Figure 10 shows the mechanical model of the expansion deformation of TWIP steel [26]. Two assumptions are made in the analysis of mechanical properties: (1) The isotropy assumption, i.e., the mechanical properties of the expansion tube material are the same in all directions. (2) The uniformity assumption, i.e., the expansion tube material and the stress are evenly distributed.

As can be seen from Figure 10, compared with unidirectional tensile deformation, the stress state of expansion deformation is more complex. The main stress states are axial and circumferential tension and radial compression [27,28]. Stress in three directions results in a stress state coefficient that differs significantly from that of uniaxial tension. The stress state coefficient reflects the impact of the material on plastic deformation under a certain stress state. A smaller stress state coefficient indicates that it is more difficult for the material to undergo plastic deformation under that stress state. The formula for the stress state coefficient was obtained from the literature:

(6) $$\beta =\frac{\sigma_{1}-\sigma_{3}}{2\sigma_{1}-0.5\left(\sigma_{2}+\sigma_{3}\right)}$$

From the balanced force equation, the expressions of axial force $\sigma_{l}$, circumferential force $\sigma_{\theta }$, and radial pressure $\sigma_{r}$ can be obtained. The mathematical relationships of the three forces are quoted from the literature [29] as:

(7) $$\sigma_{l}=\mu \sigma_{r}$$

(8) $$\sigma_{r}=\sigma_{s}\frac{t}{r}\cos\alpha \{1-\lambda [1-{\left(\frac{r}{r_{1}}\right)}^{D}]\}$$

(9) $$\sigma_{\theta }=\sigma_{s}\{1-\lambda [1-{\left(\frac{r}{r_{1}}\right)}^{D}]\}$$

In these equations, $\lambda =\frac{\left(1+D\right)}{D}$, D=μcot⁡α,$\;\lambda =\frac{\left(1+D\right)}{D}$, D=μcot⁡α, *t* is the wall thickness of the expansion tube, *r* is the radius of the lower half of the expansion tube, *r*_1_ is the radius of the upper half of the expansion tube, *α* is the expansion tube, and *μ* is the friction coefficient between the expansion cone and the inner wall of the pipe.

$\sigma_{\theta },\sigma_{r},{\;\mathrm{and}\;\sigma }_{l}$ are substituted for $\sigma_{1},\sigma_{2},{\;\mathrm{and}\;\sigma }_{3}$ in Equation (10), respectively, to obtain the final stress state coefficient of expansion deformation:

(10) $$\beta =\frac{1-\mu \frac{t}{r}\cos\alpha }{2+0.5\times \left(1-\mu \right)\frac{t}{r}\cos\alpha }$$

From the formula, it can be found that the stress state coefficient of the expansion force is related to the radius of the expansion tube, the wall thickness of the expansion tube, the expansion cone angle, and the friction factor between the expansion cone and the expansion tube. It is evident from this formula that the coefficient of the stress state of the expansion force is smaller than the coefficient of the stress state of uniaxial tension (0.5). This indicates that expansion deformation is not conducive to the plastic deformation of the material, causing the suppression of expansion plastic deformation in TWIP steel, an increase in material brittleness, and a decrease in the expansion strain rate.

### 3.5. Mechanism Discussion

**Figure 11:**
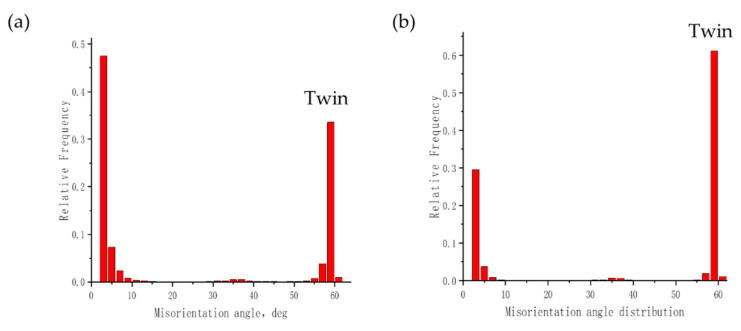
Twin integral fractions of uniaxial tensile fracture and expansion fracture of TWIP-Ⅰ steel: (**a**) uniaxial tensile fracture; (**b**) expansion fracture.

The results of the mechanical analysis in the previous section provide macroscopic data for understanding the microscopic deformation mechanism of TWIP steel during the expansion process. The difference between expansion deformation and tensile deformation leads to the difference in the micromorphology of the twins. Figure 11 shows the proportion of twin boundaries in TWIP steel after tensile and expansion fractures, as obtained via EBSD testing. It can be observed that the twin crystal fraction in TWIP steel after tensile fracture is 0.33, while the twin crystal fraction after expansion fracture is as high as 0.6. This indicates that the formation of twins is promoted by the highly constrained deformation and small stress state coefficient during the expansion process, resulting in an increased density of twins. The high twin density is also the direct cause of the low plasticity of the expansion deformation in TWIP steel.

**Figure 12:**
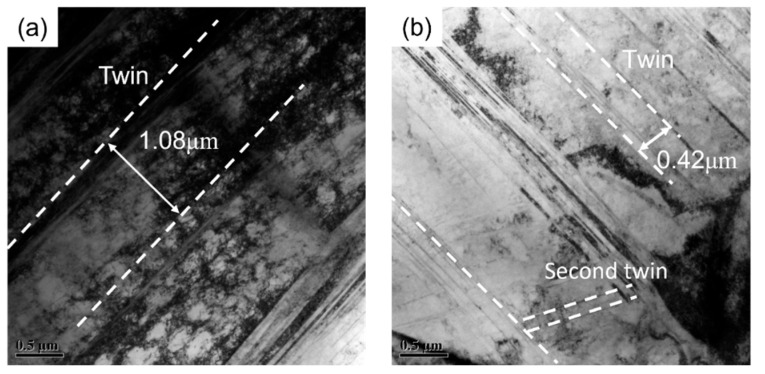
Microstructure of uniaxial tension and expansion fractures of TWIP-I steel via transmission electron microscopy: (**a**) uniaxial tension fracture; (**b**) expansion fracture.

One manifestation of the increase in twin density during expansion and deformation in the micromorphology is the dense arrangement of the twin planes. From the twin morphology observed via transmission electron microscopy, shown in Figure 12, it is clear that the distance between adjacent twin planes after tensile deformation fracture is 1.08 μm, while the distance between twin planes after expansion fracture is 0.42 μm. This shows that under expansion deformation conditions, the arrangement of twin planes in TWIP steel is denser than that under tensile deformation conditions, and the distance between the secondary twin planes produced is lower. The close arrangement of twin planes leads to a reduction in the free path of dislocation slips during the expansion of TWIP steel. This restriction of dislocation slips during expansion deformation results in a decrease in the plasticity of TWIP steel.

**Figure 13:**
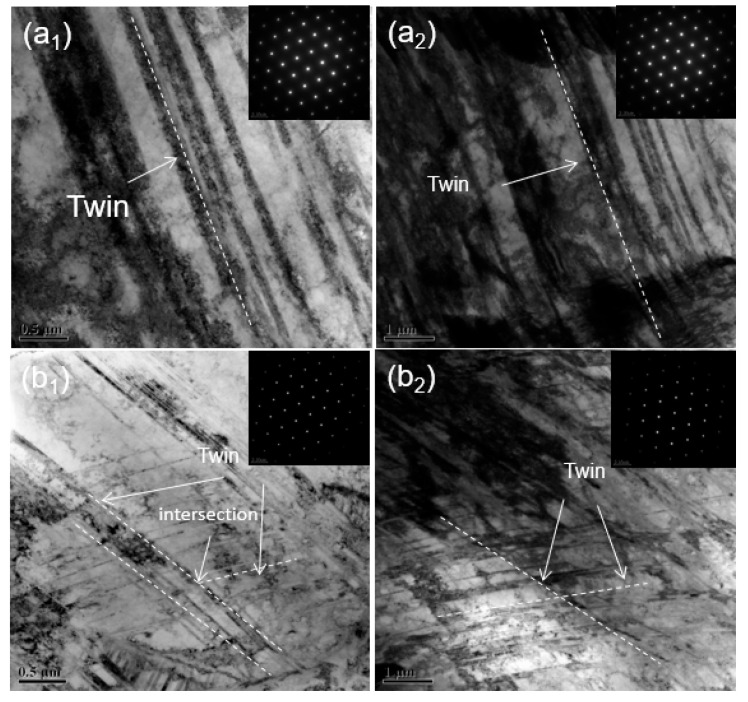
Transmission structure of TWIP steel under uniaxial tensile and expansion deformation: (**a**) uniaxial tensile deformation, (**a_1_**,**a_2_**) are different positions; (**b**) expansion deformation, (**b_1_**,**b_2_**) are different positions.

Another manifestation of the increase in twin density during expansion and deformation is the formation of a large number of secondary twins and, at the same time, a large number of intersections with primary twins. Generally, the twins generated during TWIP steel deformation play a role in promoting the strength and plasticity of the material because these twin boundaries help refine the grains. This fragment describes the dynamic Hall–Page effect in TWIP steel. However, due to the complex stress state, this highly constrained deformation promotes the formation of twins. The large number of twins formed thereby greatly limits dislocation slippage during the deformation of the material, and at the same time, the accumulation of dislocations increases. Figure 13 shows the different morphologies of tensile deformation and expansion deformation twins. Using transmission electron microscopy, it was observed that the twin delivery after expansion and deformation is very dense and the shape of the delivery is slightly different, with some twin deliveries exhibiting a penetration phenomenon.

**Figure 14:**
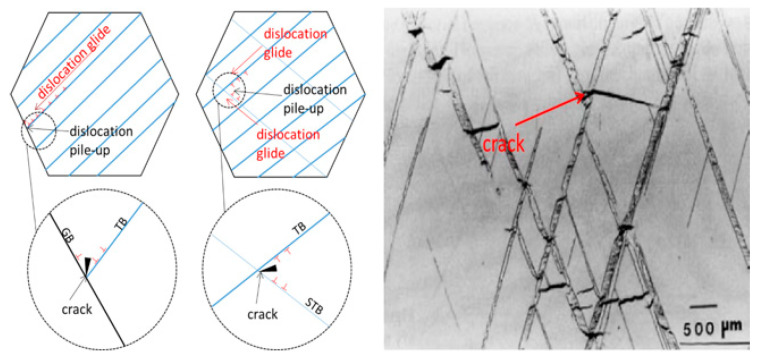
Dislocation slip and crack generation in expansion deformation of TWIP-I steel.

The microscopic twin configuration in TWIP steel during the expansion process is the reason for its low plasticity; the influence of twin morphology on plasticity is mainly realized through the joint action of twin boundaries and dislocations. In the case of uniaxial tensile fracture, the configuration of twins is a single parallel primary twin; after expansion fracture, the twin configuration is a large number of primary twins and secondary twins, and the adjacent twins are arranged more densely. When dislocations slip along the twins, due to the obstruction of twin delivery, the dislocation slip stops here, resulting in more serious accumulation of dislocations at the delivery point. At the same time, the development rate of twins is quite fast. During the process, collisions will occur between twins produced by expansion and deformation. A large number of dislocations begin to accumulate at the intersection of twins, so large residual stress forms here, and due to the decrease in the free path of dislocation slip caused by the dense arrangement of twins, the accumulation of dislocations will become more severe. The rapid and severe stress at the intersection of twins will lead to material fracture due to the continuous accumulation. In addition, the violent collision of twins here will greatly increase the stress concentration at the intersection of twins, which will lead to expansion behavior. This is the main reason for the decrease in plastic deformation ability. It can be seen from Figure 14 that there are a large number of crack sources at the intersection of twins. The slip of dislocations in TWIP-I steel is hindered by the twins. A large number of dislocation accumulations occurs at the intersection of twins, resulting in stress concentration. At the same time, due to the obstruction of the growth of twins caused by twin collisions, a large amount of stress is released at the collision, which causes the initiation of crack sources at the intersection of twins and is also the source of crack expansion [30].

## 4. Conclusions

In this experimental study, the microstructure of TWIP steel after uniaxial tensile and expansion deformation was compared through different observation methods, and the low plastic behavior of TWIP steel after expansion was explained. It was found in the experiments that there are large differences in the twin morphologies in TWIP steel under the two deformation modes of expansion and tension. The low plasticity of the expansion deformation of TWIP steel can be explained from macroscale and microscale perspectives. The conclusions are as follows:

1. Through an analysis of the expansion mechanical model, it was found that, compared with uniaxial stretching, expansion deformation is a multi-directional, force-bearing, high-constraint, and high-strain deformation mode. The stress state coefficient of this deformation mode is lower. This causes TWIP steel to exhibit lower plasticity during expansion deformation, and is the macromechanical reason for this phenomenon.
2. The morphology of the twins during the expansion deformation of TWIP steel is different from that of the twins during uniaxial tension. This difference leads to the low plastic behavior of TWIP steel during expansion deformation. From a comparison of expansion and tensile deformation, it was shown that the former produces more twins. Due to the emergence of a large number of twins, the number of twin boundaries increases, leading to delivery between twins, which hinders dislocation slips during the expansion deformation process. A large number of dislocations accumulate and a large amount of stress is concentrated at the intersection of twins, which in turn lead to crack formation and expansion during the expansion and deformation of TWIP steel, resulting in a decrease in the plasticity of TWIP steel.

The low plastic behavior of TWIP steel during expansion deformation is the biggest obstacle limiting its application in expandable pipe materials. This research shows that, although the unique deformation mechanism of this material can provide a high strength and plasticity under unidirectional force, under multi-directional force deformation conditions, such as expansion deformation, its plasticity will also be reduced by the deformation mechanism.

## Figures and Tables

**Table 1:** Chemical composition of the steel used in experiments.

Element	Mn	Si	Al	C	Fe
(wt.%)	24.4	0.90	1.82	0.21	Remainder

## Data Availability

No new data were created or analyzed in this study. Data sharing is not applicable to this article.
